# What Are Medicines; What the Distinctions between Food and Medicines; Their Classification for Therapeutic Purposes; Ætiology

**Published:** 1875-03-01

**Authors:** N. S. Davis


MEDICAL EXAMINER.

A

*Semi-Monthly Journal of Medical Sciences.*

EDITED BY N. S. DAVIS, M.D., AND F. H. DAVIS, M.D.

No. V. CHICAGO, MARCH 1, 1875. VOL. XVI.

### Original Communications.

### WHAT ARE MEDICINES; WHAT THE DISTINCTIONS BETWEEN FOOD AND MEDICINES; THEIR CLASSIFICATION FOR THERAPEUTIC PURPOSES; ÆTIOLOGY.

A LECTURE IN THE REGULAR COURSE ON PRACTICAL MEDICINE, IN CHICAGO MEDICAL COLLEGE, BY N. S. DAVIS, M.D.

HAVING in the preceding lectures endeavored to explain, as familiarly as possible, the nature and conditions of health and disease in the living human body, I must now direct your attention to some thoughts on the nature and modus operandi of medicines. Remedial agents and influences properly embrace everything that can be made useful in alleviating or curing disease.

In this sense, an encouraging word or cheerful look is as much a remedial agent as a pill or a powder from the apothecary. It is my intention, however, to limit your attention during the present hour to those material agents ordinarily styled medicines, reserving the consideration of other influences for another occasion. Medicines, in this restricted sense, are such agents as are capable of being introduced into the living system, and exerting a modifying influence over one or more of the properties or functions of the body, without being capable of assimilation or addition, as nutritive matter, to any of the tissues. They may be introduced into the system through the digestive organs, through the lungs by inhalation, through the skin by absorption, through the subcutaneous tissue by hypodermic injections, and by injection directly into the blood-vessels. The first is the more common and practically important method. But in whatever way the medicine is given it enters the mass of the blood generally unchanged in its composition, and induces its effects by passing with the blood into contact with the various structures of the body, and by such contact modifying either the properties or molecular changes, or both, in one or more of these structures. As a general rule, they are also sooner or later eliminated from the blood by some of the excretory organs in so nearly the same condition as when they were introduced, that they can be readily detected by the proper chemical tests. The apparent exceptions to these rules are such alkalies and alkaline earths as are capable of uniting directly with and neutralizing acids in the stomach before time for absorption.

Even in these cases, however, the resulting compound is absorbed, and after passing the round of the circulation, is eliminated. The real exceptions are those agents that act directly on the structures to which they are applied, as in the case of sinapisms, blisters, caustics, etc. The disposition on the part of many writers to call all the hydro-carbonaceous substances respiratory food, and those substances that simply retard the process of disintegration *indirect* food, has caused some confusion in regard to the distinction between food and medicine. It seems to me, however, that there are two plain and essential points of difference between these two classes of substances.

The first is, that food proper never passes through the digestive and assimilative organs without important changes in composition and form, and never re-appears in the excretions from either skin, kidneys, or lungs, in the same form as it entered the system. For instance, the principal proximate elements of our food are starch, gum, sugar or glucose, fat or oils, gluten, casein, the fibrin and albumen of flesh, and the inorganic salts with which they are united. If you note carefully the successive changes of the food into chyme, chyle, lacteal fluid, etc., you will find all these proximate elements radically changed before they reach the mass of the arterial blood. And you will seek in vain for any one of them in the eliminations from the true excretory organs of the body. This is directly opposite to what I have represented to be the behavior of medicine in passing through the system.

The second distinction is, that food taken in a healthy state of the system, always satiates the appetite for the time being, and that too in about the same quantity without regard to the length of time it may have been used. For instance, if a person eats bread three times a day for twenty years, he is just as readily satisfied at the end of the time as he was at the beginning. Natural appetite or hunger is simply the demand for material to supply the waste of tissue, and every substance capable of assimilation, when taken, will satisfy that demand; and with that satisfaction ceases, for the time being, all relish for more.

No such effect, however, will follow from the taking of materials that cannot be assimilated and added to the tissues by nutrition. Hence, daily observation shows that all those excitants, like the active principles of tea and coffee, and the anæsthetics, or retarders of tissue disintegration or waste, like alcohol, ether, chloroform, tobacco, etc., which have been classed by some as *indirect* food, have no power to satisfy except by mechanical fullness of the stomach, or by such a degree of stupor or anæsthesia as renders the individual for the time oblivious to further impressions.

And all these articles, instead of producing the same effects in the same quantities for any number of years, as is the case with real food, invariably create a steadily increasing demand for more. You see the young lady who sipped daintily a cup of drink at her breakfast, containing a tablespoonful of tea or coffee diluted with milk and water, ten or fifteen years later in life taking two cups at the same meal, each filled, not with milk and water flavored with a tablespoonful of tea, but with a strong infusion of tea or coffee.

In like manner, you see the young man at 18 years taking but one cigar and one glass of beer or wine per day; at 30 years consuming five or six cigars and as many drinks of beer, with now and then a glass of distilled spirits; and at 40 years he consumes a dozen cigars a day, and the number of drinks is limited only by the quantity required to induce intoxication. It is true that most, if not all, of these agents used habitually induce such a morbid condition of the stomach as to impair or destroy the appetite for proper food, but not for themselves. On the contrary, the latter grows stronger and stronger, more and more insatiable, until it too often becomes the ruling despot of the individual's life.

Having thus defined what is meant by medicines, as distinguished from food or aliments, I will divide the whole into two great classes, as follows:

First, Those substances that are capable, by their presence in the blood, of modifying the properties common to all the tissues, in such a way as to produce a perceptible change in one or more of the general processes taking place in the living body. These may be called general remedies, because in modifying the general processes of nutrition, disintegration, and calorification, they necessarily influence in some degree all the functions of the animal economy.

Second, Those substances which, though introduced into the mass of the blood, exhibit an affinity for, or special action on, some particular organ or group of organs, and hence may be termed local remedies.

The remedies included in the first class may be arranged in four groups, called general stimulants, or excitants; general tonics; general sedatives; and general alteratives.

The first group embraces those substances that increase or exalt the susceptibility of the tissues simply, as tea, coffee, caloric, oxygen, etc.

The second includes such agents as are capable of increasing the play of vital affinity, either alone or in connection with a moderate increase of susceptibility, thereby giving an increased tonicity to the structures of the body, and generally an increase in the evolution of caloric. To this group belong the preparations of iron, the mineral acids, guaiac, cantharides, phosphorus, many of the bitter vegetable alkaloids, etc.

The third group embraces those agents that are capable of influencing either the susceptibility, or the vital affinity, or both, in the opposite direction from either of the preceding groups. That is, they either depress the susceptibility or impair the play of vital affinity, or both at the same time. Consequently they diminish the molecular changes constituting nutrition, disintegration and secretion, and diminish both the evolution of caloric and the capacity to receive impressions. To this group belong the hydrocyanic and carbonic acids, the alkalies, the bromides, alcohol, ether, chloroform, etc. Some of you may be surprised to see the alcoholic liquids included among the general sedatives. But all the experiments with alcohol, from the days of Dr. Prout to the present time, have shown that while present in the blood it directly diminishes the temperature of the tissues, retards the atomic changes and the amount of eliminations, and diminishes the general susceptibility. If these effects do not constitute it a general organic sedative, it would be difficult to conceive what should be ranked as such.

The fourth group embraces such agents as are capable of modifying the properties of the tissues in a manner different from that of simple increase or diminution, and hence they are called alteratives. To this group belong iodine, mercury, arsenic, and their several preparations, together with those agents that are supposed to neutralize poisons in the blood, or to prevent what are called zymotic changes in that fluid, such as the sulphites, permanganates, carbolic acid, etc.

These definitions are sufficient to give you a correct idea of what I mean by *general* remedies.

They produce their effects by acting on those elementary properties that are common to all the structures of the body. When they increase or exalt these properties, they are stimulants and tonics; when they impair or depress, they are sedatives; when they modify the properties different from either increase or diminution, they are alteratives.

But much the larger part of the remedies in the works on materia medica belong to the second class, called local remedies. Quite a number even of those I have enumerated as general remedies will be found to possess, in addition, a direct local influence on some structure or organ. Thus alcohol, by its presence in the blood, not only retards molecular changes throughout all the tissues as a general remedy, but like all true anæsthetics, it also diminishes locally the sensibility of the cerebro-spinal nervous centres. So, too, the tea and coffee, which have been ranked as general excitants, are capable of exerting a special local influence over certain portions of the nervous system, inducing wakefulness, palpitations, muscular tremors, etc.

The group of remedies usually styled narcotics or soporifics act more exclusively upon the brain and nervous centres. They directly diminish the sensibility of the nerve structures, and thereby relieve pain and favor sleep. In large doses they are capable of so completely suspending cerebral sensibility as to cause coma and death. To this group belong opium, conium, hyoscyamus, lactuca, chloral, etc. Though all these agents diminish cerebral sensibility, they do not all act alike. Thus opium and its preparations cause dilatation of the smaller vessels of the nervous centres, and consequently increased accumulation of blood; while hyoscyamus, belladonna and stramonii cause contraction of the vessels, and thereby lessen the quantity of blood in the part. The action of the former is accompanied by contraction of the pupils of the eyes, the latter by dilatation. Although the narcotics act primarily on the nervous tissues, yet, by diminishing nerve sensibility, they secondarily diminish the influence of the nervous over the muscular structures, and thereby impair the respiratory movements, circulation, the peristaltic motion of the bowels, and to some extent the action of muscles of voluntary motion.

Another class of agents, when introduced into the system, are capable of so modifying the circulation and properties in the cutaneous tissue as to cause a marked increase in the amount of exhalation from the surface, and you call them diaphoretics or sudorifics. Another class exert a similar influence on the mucous membrane of the respiratory passages, and you call them expectorants. Another class so influence the kidneys as to increase the quantity of urine secreted in a given time, and they are called diuretics. Still another class so modify the condition of the mucous membrane of the stomach and bowels, and quicken the peristaltic motion, as to result in increased gastric and intestinal discharges, and they are called emetics and cathartics. The last four groups of remedies so alter the play of affinity in the organs on which they act as to increase secretory action.

But there are remedies acting on the several organs in the opposite direction; that is, in such a manner as to diminish secretion, and they are called astringents. Again, we have remedies of more or less value that do not directly modify either the structure or function of any part of the system, but which exert a purely chemical or mechanical influence. Thus you may give acids to neutralize an excess of alkalies, either in the stomach or the blood; or alkalies to neutralize an excess of acids. Pepsin, hydrochloric acid, and many other substances may be used as gastric solvents when the natural gastric juice is deficient.

There are also remedies of great value that do not properly belong either to the group of general organic sedatives or to the local narcotics. When properly administered they are capable of either diminishing the action of the heart and arteries, or of lessening the excitability of the cerebro-spinal and vaso-motor nervous centres. Those that appear to directly diminish the action of the heart and arteries, as the veratrum viride, aconite, digitalis, cold, and venesection, may be called vascular sedatives. Those that more prominently diminish the excitability of certain portions of the nervous apparatus, as the gelsemium, calabar bean, ergot, cimicifuga, etc., may be styled nervous sedatives.

In thus glancing rapidly over a therapeutic arrangement of remedial agents, it is no part of my purpose to enter upon the discussion of the modus operandi of medicines, but simply to give an outline of such a classification as would correspond with the views expressed in the preceding lectures concerning the nature and varieties of disease.

If an accurate knowledge of physiology, or the conditions of function and structure which constitute health, is essential as a starting-point for acquiring a knowledge of disease, so is a thorough study of the nature and modus operandi of medicines essential as a preparation for their intelligent application in the treatment of disease.

It is to be hoped, therefore, that all of you have given due attention to this branch of medical science during the earlier part of your studies.

If not, I cannot too strongly urge upon you the importance of early supplying the deficiency.

To note down formulas or prescriptions and apply them in the treatment of particular diseases, simply because they were recommended by your teachers, without an accurate knowledge of each of their constituents and the special effect it is expected to produce, is to exhibit a blind dependence on authority degrading to the practitioner and dangerous to his patients. The conditions essential for the rational practice of medicine are: a clear conception of the morbid conditions affecting the patient, an equally reliable knowledge of the nature and action of medicines, and the discipline of mind necessary for accurately adjusting the latter to the fulfillment of the indications presented by the former.

The general indications for the employment of remedial agents have been variously classified by different writers. The most simple and convenient arrangement is to consider first, those having for their object the removal of the cause or causes; second, those arising from the essential pathology of the disease; and third, those afforded by the secondary symptoms or consequences of the primary pathological condition.

There are many morbid conditions which speedily cease by simply removing the causes that have induced them. There are others which, when fairly commenced, continue, though generally with less activity, after the causes have wholly ceased to act. Hence, a correct knowledge of the nature and modus operandi of the causes capable of giving rise to particular forms of disease, is of great importance both to the physician and the community. Such knowledge not only enables the physician to treat individual cases of disease more successfully, but it enables both individuals and communities to adopt such sanitary and hygienic measures as to greatly lessen the prevalence and fatality of many of the most important diseases to which our race is subject.

Ætiology, therefore, affords the only reliable foundation for the sanitary improvement of cities, populous towns, and even rural districts. To this department of medical science the world is indebted for all the advantages it derives from systems of sewerage, scavengering, water supply, modes of ventilation, improved construction of dwellings, etc.

And yet there is no field in which more careful and patient labor is needed, or which will yield the laborer a richer reward. For though very much has been accomplished in ascertaining the special circumstances which favor the development of many morbid causes, the laws that govern their diffusion, and their influence on the human system, yet but little progress has been made in the work of isolating and studying the nature, composition and properties of the several causes themselves. What has already been accomplished affords a broad foundation for most important sanitary improvements, both of an individual and municipal character; but what remains to be done in this department would add much to this foundation, and still more to our success in endeavoring to remove the causes acting injuriously on our individual patients. Diseases are often produced by the joint action of several causes, some of which act with feeble intensity but continuously through considerable periods of time. Others act more abruptly and with greater intensity. The first usually produce their effects slowly, merely modifying slightly the properties of the tissues, or the functions of one or more organs, without at once developing the phenomena of active morbid action, and hence are called remote or predisposing causes. The second, acting with more intensity, and more directly developing marked symptoms of disease, are called exciting causes. The division, however, is an artificial one, as nearly all the predisposing causes become direct exciting ones by simply increasing their intensity or prolonging their duration. A more natural division of causes would be into external and internal. The first embracing all agents and influences originating exterior to the living body, and capable of making an unnatural impression upon any of its parts, and the second such as originate within the living organization. The external causes are resolvable into two classes, viz.: such as consist in changes either in composition, quality or quantity, of the natural ingesta, including under this latter term the air, water and food, and such as consist of agents not belonging to the natural ingesta, but capable of being received into the system through the same channels. I need hardly remind you that the air we breathe is composed of nitrogen, oxygen, ozone or active oxygen, carbonic acid, caloric, and electricity, and that these constituents are subject to almost constant variations. When such variations do not exceed certain limits they are consistent with a continuance of health in the animal economy; but when they are too abrupt or extreme, they are productive of morbid conditions often of the most dangerous character. The atmospheric elements most subject to such extreme changes are the ozone, caloric and electricity.

The most superficial observer knows that there is habitually a wide difference between the character of diseases prevailing during the high temperature of summer and the low temperature of winter. The influence of caloric in increasing the susceptibility and lessening the vital affinity or tonicity of living structures is as apparent as its power to lessen the affinity by which the atoms of inorganic matter are held together. Many facts point to an intimate relation between extreme changes in the ozone, caloric, electricity and aqueous vapor of the atmosphere, and the prevalence of such diseases as influenza, catarrh, rheumatism, cholera, etc., and there is much need of further careful investigation in this direction.

The second division of the external causes of disease, consisting of agents not naturally entering into the composition of air, water, or food, but capable of being mixed with one or more of these, and imbibed into the system through the same channels, arise mostly from the disintegration or decay of dead organic matter, both animal and vegetable.

Hence, from a remote period in the history of medicine, they have been styled *miasms*. Idio-miasms, when the product of the decomposition of animal matter or animal excretions, and koino-miasms, when from the decomposition of vegetable matter. Until a comparatively recent period, these deleterious products were very generally regarded as inorganic, gaseous, or chemical compounds, although so subtle and attenuated as to be ever eluding the best directed efforts of the chemist to isolate and examine them. By a few, however, like Copeland and Holland in Europe, and J. K. Mitchell in America, they were regarded as organic living germs, either vegetable or animal—fungi or animalculæ. And since the general use of the microscope in medical investigations, the tendency to regard all the deleterious products of the decomposition of organic matter as organized microscopic germs, capable of self-propagation, and free diffusion in connection with the aqueous vapor of the atmosphere, has greatly increased throughout all ranks of the profession. Still, there is very little agreement among the various observers, and the whole subject needs much more extended and patient investigation.

In all our researches with the microscope, concerning the nature of morbid causes, great care is required lest we mistake the mere products or results of morbid action for the causes. For instance, if we examine the surface of a sore on the skin, or of an ulcer in the mucous membrane of the mouth, and find it covered with fungi or vegetable germs, it does not necessarily follow that such fungi were the cause of the diseased spots in either place. Neither does it follow as a legitimate deduction that certain violent epidemic diseases, as cholera for example, arise from organic germs merely because such have been seen in the excretions. To furnish data for any legitimate deductions from this class of observations, they must be made at the very incipient indications of disease, and repeated at the different stages of its progress, and after full recovery. Similar observations must also be made during the progress of other diseases affecting the same organs or structures. The first series of observations are necessary to determine whether the supposed germs are always present in a given disease, and whether present in all stages of its progress, or only at certain periods. The second series are necessary to determine whether they are peculiar to the one disease, or present in many and dissimilar diseases. One observer places a few specimens of cholera evacuations on the microscopic field, and observing certain organic germs, which, on keeping a certain length of time, develop into a species of fungus, and he straightway announces the discovery of the direct cause of cholera. Another places a specimen of the blood of a syphilitic patient on the microscopic field, and after patiently watching it for two or three days, a crop of living bodies make their appearance, and we have another grand pathological discovery.

But just as the literature of the profession has become well filled with the important discoveries, and the many practical applications of which they are capable, behold, some one else has also discovered that the special cholera fungus can be found as well in any serous intestinal evacuation, and that the so-called syphilitic germs are easily found in the blood of persons who never dreamed of having had that disease, either hereditary or acquired. It is thus that one set of investigators are constantly employed in correcting the errors of another class, and our literature is kept full of contradictory statements.

Hasty generalization, or the deduction of important conclusions from imperfect and inadequate data, has ever been one of the greatest hindrances to genuine progress, in both the science and the art of medicine.

The second class of causes, those that originate in the living system, may be called mental and physical. That either deficient or excessive mental exercise, and either sudden or long-continued action of the emotions and passions, are capable of inducing morbid action in the physical structures of the body, is too well known to need illustration. That the physical processes of metamorphosis and disintegration may be so imperfect or perverted as to cause unnatural products to be returned into the blood, or that the secretory action of one or more secretory organs may be so perverted as to cause the secretion to be unnatural in quantity or quality, and thereby become a cause of irritation and derangement, is equally obvious. This simple and hasty glance at the subject of ætiology, will be sufficient to show you both the importance of the indication for removing the causes of disease, and the practical difficulties in the way of fulfilling it.

I stated that the second indication for the use of remedies in remedial practice was founded on the nature of the disease. For instance, if the nature of the disease is such as to present increased activity and excitement, it indicates the use of soothing and sedative remedies; if increased sensibility and suffering, either narcotics or anæsthetics; if impaired activity and relaxation, excitants and tonics; if perverted vital affinity, alteratives, etc. I need not tell you that a very large part of the skill and success of the practitioner will depend on the clearness of his appreciation of the nature of the morbid actions involved in any given case, and the accuracy with which he adjusts the remedial agents to meet the indications afforded thereby.

The third indication was founded on the secondary effects of the primary disease, and the complications that supervene during its progress.

The several organs and functions of the human body are so intimately connected with, and dependent on, each other, that it is almost impossible to have disease invade one without soon causing disturbance in others. And there are few individuals who do not have a greater susceptibility in some organs than in others, and hence, when attacked by general diseases, such sensitiveness often becomes so increased as to constitute active local disease. And it often happens that the secondary affections become the most dangerous to the life of the patient. This is illustrated strikingly when any one of the principal excretory organs are involved.

Thus disease of the kidneys may be of such a nature as to prevent a proper elimination of urea, which, being retained in the blood, poisons the cerebro-spinal nervous system, producing convulsions, coma and death. Or valvular disease of the heart, by keeping up for a long time irregular circulation of the blood, causes general derangement of secretion, especially scantiness of urine and general dropsy.

The practitioner, therefore, should study carefully the mutual relation and dependencies of one function on another, that he may be prepared not only to treat the secondary derangements when they occur, but to enable him often to anticipate their occurrence by appropriate preventive measures.

All details concerning ætiology, pathology, diagnosis and therapeutic action of medicine will be given in connection with the consideration of particular diseases and groups of diseases, my present object being simply to give you such an outline as would challenge your attention, and systematize your thoughts in relation to these topics.

